# A case of pulmonary adenocarcinoma showing rapid progression of peritoneal dissemination after immune checkpoint inhibitor therapy

**DOI:** 10.1186/s12885-018-4549-5

**Published:** 2018-05-31

**Authors:** Taro Shinozaki, Eri Iwami, Shinnosuke Ikemura, Tatsu Matsuzaki, Takahiro Nakajima, Kazuhiko Hashimoto, Takeshi Terashima

**Affiliations:** 1 Department of Respiratory Medicine, Tokyo Dental College Ichikawa General Hospital, 5-11-13 Sugano, Ichikawa, Chiba 272-0824 Japan; 2 Department of Pathology and Laboratory Medicine, Tokyo Dental College Ichikawa General Hospital, 5-11-13 Sugano, Ichikawa, Chiba 272-0824 Japan

**Keywords:** Adenocarcinoma, Hyperprogressive disease, Immune checkpoint inhibitor, Lung cancer, Pembrolizumab, Peritoneal dissemination

## Abstract

**Background:**

Immune checkpoint inhibitors are standard treatments for non-small cell lung cancer. Unique cases with paradoxical acceleration of the disease after immunotherapy have been reported. These have been described as cases of hyperprogressive disease.

**Case presentation:**

A 76-year-old man was diagnosed with pulmonary adenocarcinoma with pleural dissemination and liver and adrenal metastases. Genomic analysis revealed neither *EGFR* mutations nor *ALK* translocations. Immunohistochemical analysis revealed a programmed death-ligand 1 tumor proportion score of 23%. Chemotherapy with carboplatin, paclitaxel, and bevacizumab resulted in Grade 3 skin eruption and disease progression. Pembrolizumab was initiated as a second-line treatment. However, peritoneal dissemination and ascites developed. The patient died 2 weeks later. The autopsy revealed widespread peritoneal dissemination and an extensive hemorrhagic infarction.

**Conclusion:**

This was a rare case of hyperprogressive disease with rapid progression of peritoneal dissemination after pembrolizumab treatment.

## Background

Immune checkpoint inhibitors (ICIs) are currently standard treatments for non-small cell lung cancer (NSCLC) [1, 2]. The unique adverse events that can arise after treatment with ICIs, including pneumonitis, colitis, and thyroiditis, are known as immune-related adverse events [3]. Recently, unique cases with paradoxical acceleration of the disease after immunotherapy have been reported [4, 5]. These have been described as cases of hyperprogressive disease. In one study, hyperprogressive disease was observed in 9% of patients treated with ICIs [4].

Herein, we report on a case of hyperprogressive disease after treatment with pembrolizumab, an ICI currently used in the treatment of NSCLC. The clinical course in this case was highly unusual as it exhibited rapid progression of peritoneal dissemination shortly after a single administration of pembrolizumab. Moreover, this is one of the first reports to document hyperprogressive disease by autopsy.

## Case presentation

**Fig. 1:**
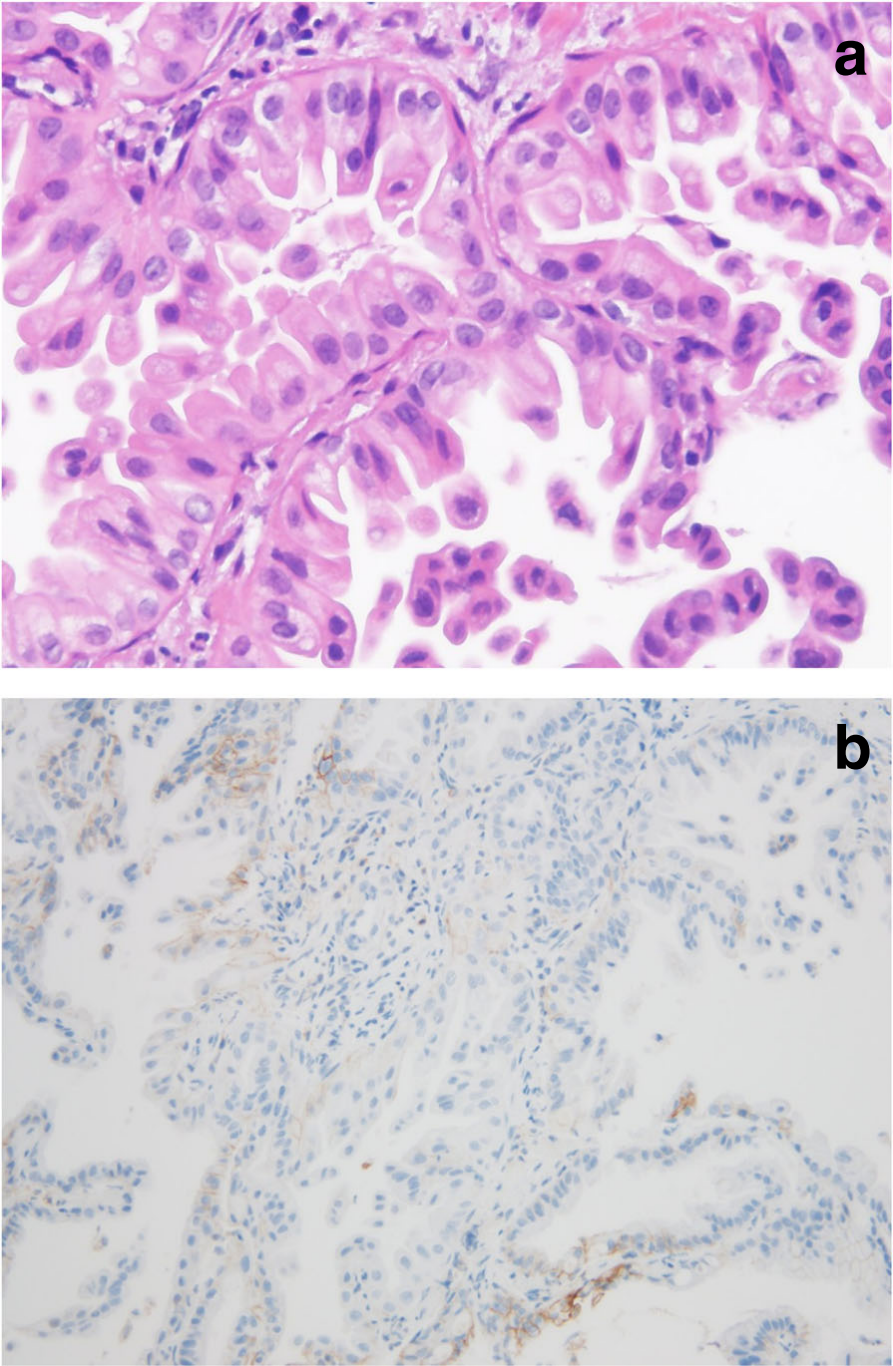
Histopathological findings of tissue specimens obtained under bronchoscopy. **a** Hematoxylin and eosin staining showing a well-differentiated adenocarcinoma (200× magnification). **b** 22C-3 antibody staining against programmed death-ligand 1. Tumor proportion score, 23%

A 76-year-old man with no history of smoking presented with an abnormal shadow on chest X-ray. He had a medical history of hypertension, dyslipidemia, reflux esophagitis, and benign prostatic hyperplasia. He had mild dyspnea on exertion. Physical examination did not reveal any abnormal findings and the results of the laboratory tests were within normal limits. The patient had an Eastern Cooperative Oncology Group performance status (ECOG PS) score of 1. Chest X-ray revealed a tumor in the left hilar region and left pleural effusion. This was confirmed by enhanced computed tomography (CT) scan, which showed a 40 mm tumor in the left lower lobe with left pleural effusion. Enhanced CT scan of the abdomen revealed liver and adrenal metastases. Tissue specimens obtained by bronchoscopy revealed a well-differentiated adenocarcinoma (Fig. 1a). Genomic analysis was performed to identify whether there were any driver mutations present. This revealed that there were no sensitizing mutations in the epidermal growth factor receptor (*EGFR*) gene, nor were there translocations in the anaplastic lymphoma kinase (*ALK*) gene. Immunohistochemical analysis of programmed death-ligand 1 (PD-L1) expression using the murine 22C-3 antibody, expressed as the tumor proportion score (TPS), revealed a TPS of 23% (Fig. 1b). Furthermore, there were no increases in the serum levels of various tumor markers.

**Fig. 2:**
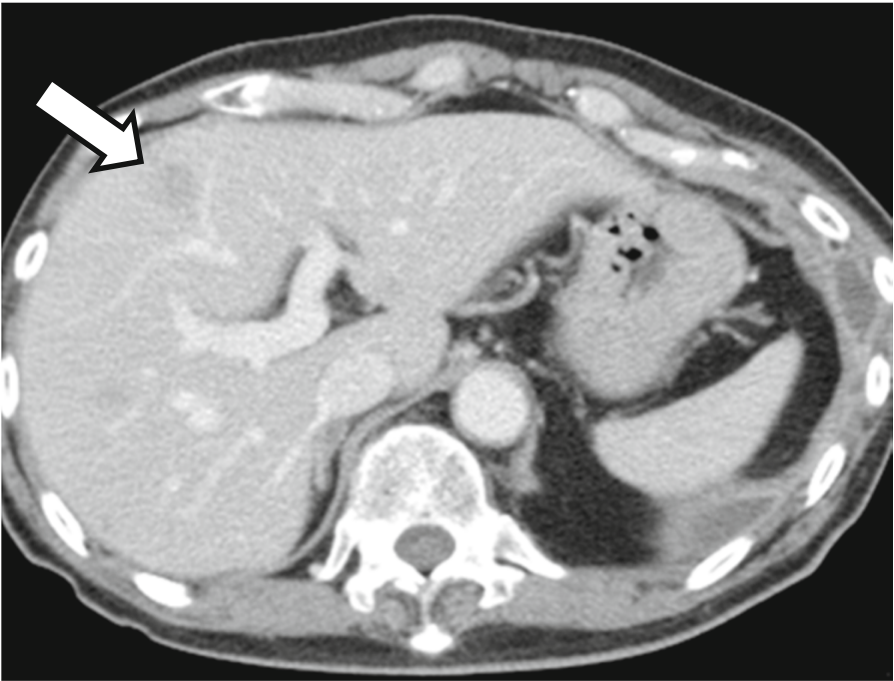
Enhanced computed tomography (CT) imaging. CT was performed after a single course of cytotoxic chemotherapy. The size of the liver metastasis in the S4 region increased from 1.0 to 1.3 cm (arrow)

**Fig. 3:**
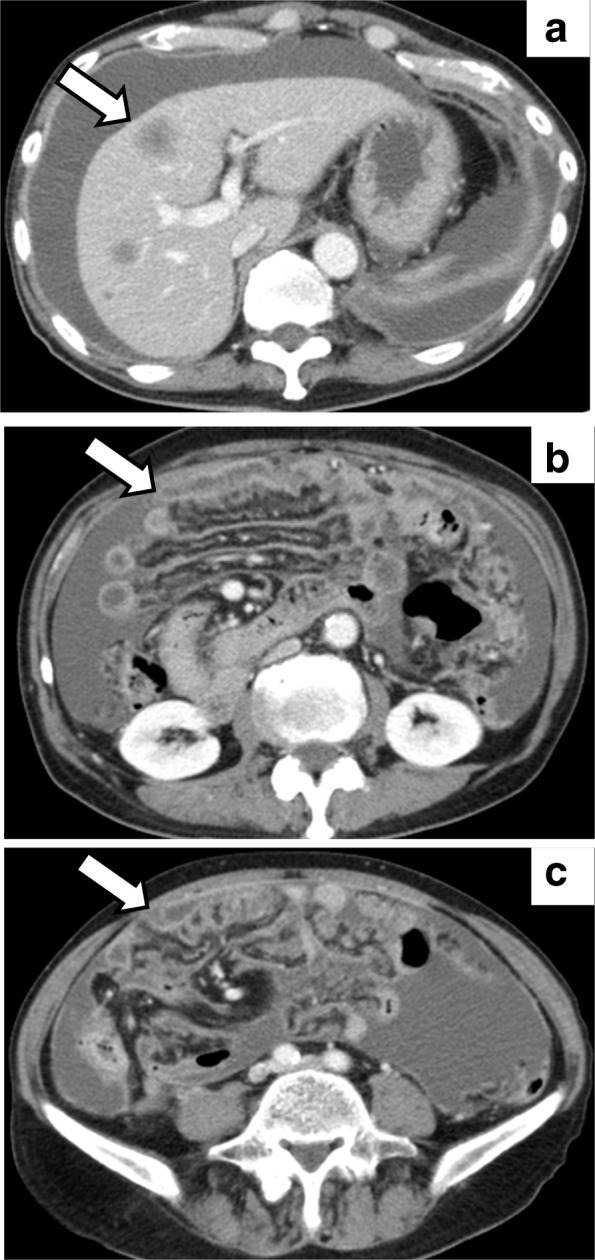
Enhanced computed tomography (CT) imaging. **a** After a single administration of pembrolizumab, the size of the liver metastasis increased from 1.3 to 1.6 cm (arrow). **b** and **c** Widespread peritoneal dissemination (arrows) and a large amount of ascites were visible after pembrolizumab treatment

First-line chemotherapy with carboplatin (area under the curve, 6), paclitaxel (200 mg/m^2^), and bevacizumab (15 mg/kg) was initiated for pulmonary adenocarcinoma cT3N1M1c (Stage IVB). However, skin eruption developed during the week following administration and spread to his whole body. The size of the liver metastasis in the S4 region also increased from 1.0 to 1.3 cm and new liver metastases were detected (Fig. 2). The ECOG PS score of the patient was 1. Since the patient’s skin eruption was classified as a Grade 3 adverse event (Common Terminology Criteria for Adverse Events, version 4.0) and the disease was progressive, treatment was switched to an ICI. Pembrolizumab (200 mg/body) was administered as a second-line treatment. Following administration, the patient began to experience a gradual loss of appetite and abdominal distension, followed by a worsening of his general condition. On Day 13 after pembrolizumab administration, an enhanced CT scan showed progression of the liver metastases (Fig. 3a). There was also a large amount of ascites and widespread peritoneal dissemination (Fig. 3b, c). These were novel findings that had not been observed prior to pembrolizumab treatment. The patient died suddenly due to rapid progression of respiratory failure on Day 14 after pembrolizumab administration.

**Fig. 4:**
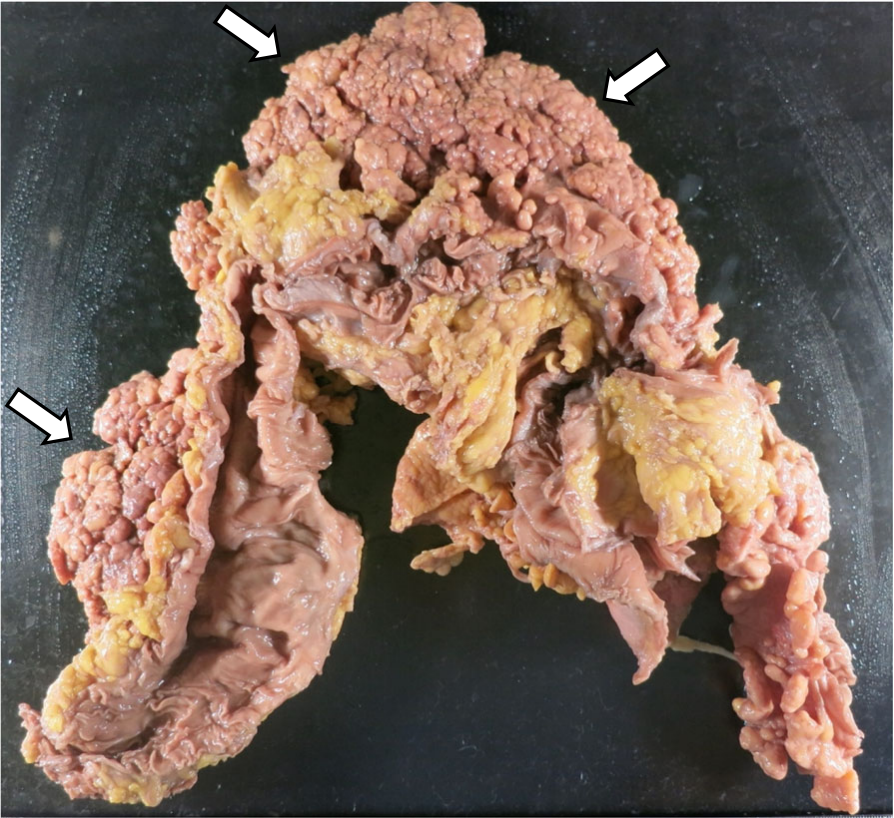
Macroscopic examination of the autopsy specimens revealed widespread peritoneal dissemination (arrows)

**Fig. 5:**
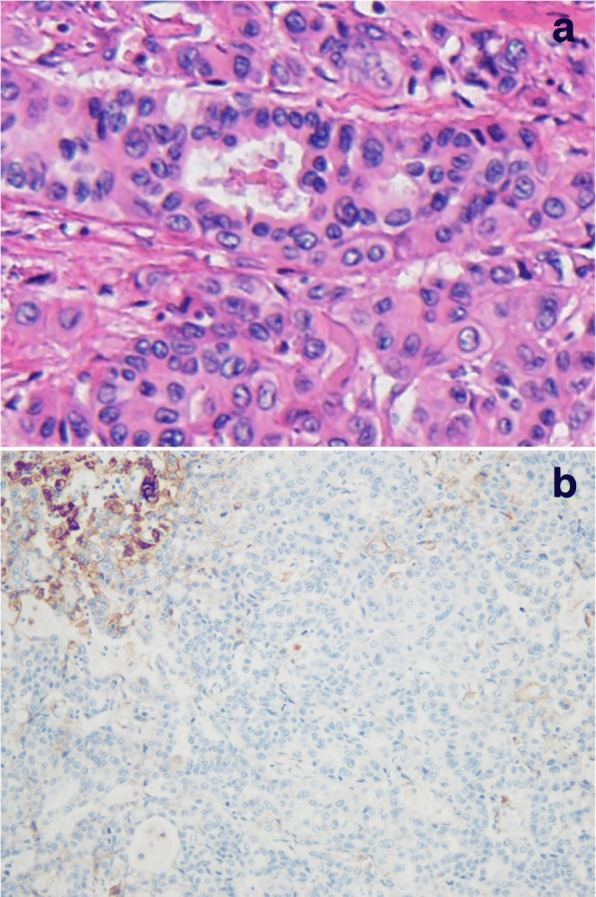
Histopathological findings of peritoneal autopsy specimens. **a** Hematoxylin and eosin staining of the peritoneal tissue revealed an invasive, well-differentiated adenocarcinoma (100× magnification). **b** 22C-3 antibody staining against programmed death-ligand 1. Tumor proportion score, 12%

**Fig. 6:**
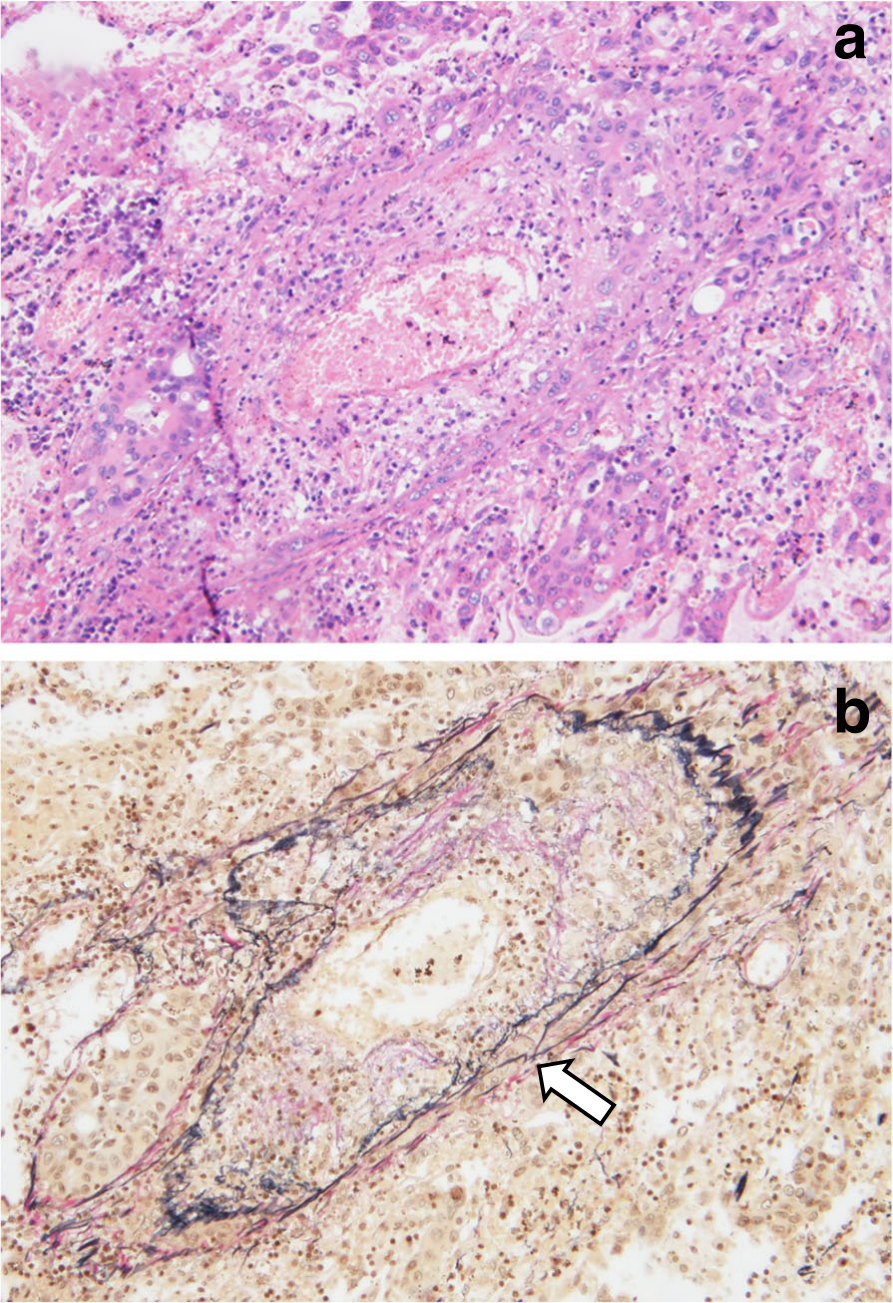
Histopathological findings of autopsy specimens from the right lung. **a** Hematoxylin and eosin staining (100× magnification). **b** Verhoeff-Van Gieson elastic staining. An extensive hemorrhagic infarction due to tumor embolism was observed (arrow)

An autopsy revealed extensive Stage IV lung adenocarcinoma originating from the left lower lobe with metastases in the lungs, left pleura, liver, adrenal glands, kidneys, pancreas, stomach, small intestine, colon, bone marrow, and lymph nodes of the bilateral hilar and para-aortic lesions. Dissemination to the peritoneum (Fig. 4), omentum, and diaphragm was also documented by autopsy. Microscopic examination of the peritoneal tissue confirmed the presence of a well-differentiated invasive adenocarcinoma (Fig. 5a). Immunohistochemical analysis of the peritoneal tissue revealed that the PD-L1 TPS of the tumor cells was 12% (Fig. 5b), which is similar to the score for the tumor tissue obtained by bronchoscopy. Overexpression of the *MDM2* gene was not detected by immunohistochemistry. In addition to these findings, an extensive hemorrhagic infarction due to tumor embolism was observed in the right lung (Fig. 6a, b). This was recorded as the cause of death based on the autopsy.

## Discussion and conclusions

To the best of our knowledge, this is the first case of lung cancer with hyperprogressive disease showing rapid progression of peritoneal dissemination after ICI treatment. Moreover, this is the first case where hyperprogressive disease was documented by autopsy.

Hyperprogressive disease has recently been described in cases treated with immunotherapy [4, 5]. In current treatment strategies for advanced NSCLC, the ICI pembrolizumab is recommended as a first-line therapy in cases where the TPS is ≥50% and as a second-line therapy in cases where the TPS is 1–49% [1, 2]. It is critical to determine whether the progression observed in this case was hyperprogressive disease, pseudoprogression, or natural progression, as is often observed in the terminal stages of malignant diseases. ICIs are sometimes known to result in unique response patterns, such as pseudoprogression [6]. However, the autopsy findings in the present case ruled out the possibility of pseudoprogression. Champiat et al. proposed that hyperprogressive disease should be defined as a > 2.0-fold increase in tumor growth rate after immunotherapy [4]. Kato et al. defined hyperprogressive disease as a time-to-treatment failure of < 2 months, a > 50.0% increase in tumor burden, and > 2.0-fold increase in tumor growth rate [5]. In our case, the scale measurable region was the liver metastases. The time elapsed between the 1.0 to 1.3 cm and 1.3 to 1.6 cm enlargement of the target lesion of the liver was 51 and 19 days, respectively. The volume doubling time before and after pembrolizumab treatment was 45 and 21 days, respectively (volume doubling time = [(T1 − T0) · log 2] / [3 log (D1 / D0)], where D1 and D0 are the diameters at T1 and T0, respectively) [7]. There was a > 2.0-fold increase in tumor growth rate since tumor growth rate is the inverse of the volume doubling time (i.e., tumor growth rate = 1 / volume doubling time) [8].

In a previous study, the median time from diagnosis of Stage IV disease to peritoneal metastasis was 16.5 (range, 0.6–108) months among 410 patients with metastatic NSCLC [9], which is notably longer than the 2.3 months in this case. Moreover, the time from pembrolizumab administration to peritoneal metastasis was just 0.4 months (13 days). The novel appearance of widespread peritoneal dissemination and a large amount of ascites within 13 days met the criteria of time-to-treatment failure of < 2 months and suggested that the clinical course of our case was much more rapid than the natural terminal course. Finally, autopsy findings revealed greater progression of the metastases than CT scan images taken 1 day prior to the patient’s death. Together, these indicate that this was a case of hyperprogressive disease.

The clinical course of our case was highly unique due to the presence of widespread peritoneal dissemination. Peritoneal dissemination is a rare clinical event in lung cancer patients, with autopsy results indicating an incidence of 9.4–15.8% [10, 11]. It is even rarer that peritoneal dissemination develops during the clinical course. A 26-year study of 1024 lung cancer patients reported that only 12 patients (1.2%) developed clinically detectable peritoneal dissemination [12]. Another study found that in 410 patients with metastatic NSCLC, 33 patients (8%) developed peritoneal dissemination and that this was highly associated with pleural dissemination [9]. In our case, it is possible that pleural dissemination and hyperprogressive disease contributed to peritoneal dissemination.

The mechanisms of hyperprogressive disease are not yet understood. Tumor profiles and patient characteristics are thought to be important factors. The finding that the PD-L1 TPS was similar before and after the administration of pembrolizumab in our case was in agreement with the finding that hyperprogressive disease was not associated with the PD-L1 status of tumors [4]. Kato et al. showed that cancer patients with *MDM2* gene amplification or *EGFR* mutations had increased rates of tumor growth after treatment with ICIs [5]. However, in our case, *EGFR* was wild-type and there was no overexpression of the *MDM2* gene. In terms of patient characteristics, age > 65 years was identified as the only associated factor for hyperprogressive disease [4]. Although there may be other unknown risk factors, it is likely that old age was the primary risk factor for progressive disease in our case.

Our report has several limitations. Firstly, there is the potential that the rapid progression seen in our patient was due to the natural course or intrinsic cancer biology rather than pembrolizumab therapy. In fact, the disease was chemoresistant and progressive prior to pembrolizumab treatment. The widespread peritoneal dissemination, however, suggested accelerated progression after pembrolizumab treatment. Secondly, the tumor growth rate is not a standardized model for response and the definition of hyperprogressive disease proposed by Champiat et al. has not been widely accepted by the broader oncology community [13]. In our case, the rapid growth of the tumor was shown by a shortening of the doubling time of the target lesion of the liver. The strength of our case is the fact that true progression was documented by autopsy and that autopsy specimens will be useful for elucidating the mechanisms of hyperprogressive disease in the future.

In this report, we describe a case of pulmonary adenocarcinoma showing rapid progression of peritoneal dissemination soon after a single administration of pembrolizumab. It is likely that old age was a risk factor for progressive disease after ICI treatment. Clinicians should consider the possibility of hyperprogressive disease in a small subset of patients after ICI treatment. Further studies are needed to elucidate the risk factors and mechanisms of hyperprogressive disease following immunotherapy.

## Abbreviations

ALK: Anaplastic lymphoma kinase
CT: Computed tomography
ECOG PS: Eastern Cooperative Oncology Group performance status
EGFR: Epidermal growth factor receptor
ICI: Immune checkpoint inhibitor
NSCLC: Non-small cell lung cancer
PD-L1: Programmed death-ligand 1
TPS: Tumor proportion score
